# Synthesis of Cycloveratrylene Macrocycles and Benzyl Oligomers Catalysed by Bentonite under Microwave/Infrared and Solvent-Free Conditions

**DOI:** 10.3390/molecules181012820

**Published:** 2013-10-16

**Authors:** René Miranda, Omar Valencia-Vázquez, Carlos Abel Maya-Vega, Inés Nicolás-Vázquez, Yolanda Marina Vargas-Rodriguez, José Antonio Morales-Serna, Eréndira García-Ríos, Manuel Salmón

**Affiliations:** 1 Departamento de Ciencias Químicas, Campo 1, Facultad de Estudios Superiores Cuautitlán, Universidad Nacional Autónoma de México, Av 1o de Mayo s/n, Sta. Ma Las Torres, Cuautitlán Izcalli 54740, Estado de México, Mexico; E-Mails: carte345@live.com (O.V.-V.); carte345@yahoo.com.mx (C.A.M.-V.); nicovain@yahoo.com.mx (I.N.-V.); ym_vargas@yahoo.com.mx (Y.M.V.-R.); 2 Instituto de Química, Universidad Nacional Autónoma de México, Circuito Exterior, Ciudad Universitaria, Coyoacán 04510, México, D.F., Mexico; E-Mails: morser@unam.mx (J.A.M.-S.); erengr@unam.mx (E.G.-R.)

**Keywords:** bentonitic clay, solvent-free, microwave-assisted, infrared irradiation, cycloveratrylene

## Abstract

Tonsil Actisil FF, which is a commercial bentonitic clay, promotes the formation of cycloveratrylene macrocycles and benzyl oligomers from the corresponding benzyl alcohols in good yields under microwave heating and infrared irradiation in the absence of solvent in both cases. The catalytic reaction is sensitive to the type of substituent on the aromatic ring. Thus, when benzyl alcohol was substituted with a methylenedioxy, two methoxy or three methoxy groups, a cyclooligomerisation process was induced. Unsubstituted, methyl and methoxy benzyl alcohols yielded linear oligomers. In addition, computational chemistry calculations were performed to establish a validated mechanistic pathway to explain the growth of the obtained linear oligomers.

## 1. Introduction

The development of general, catalytic and selective procedures for benzyl alcohol C-O bond activation is highly desirable and would constitute a broadly applicable set of transformations in organic synthesis [1]. Because a benzyl alcohol C-O bond is more reactive than that of an aliphatic alcohol, benzyl alcohol C-O activation is favoured in acid-catalysed reactions because of the greater stability of the intermediate formed [2,3]. This activation reaction has been used as a strategy in the synthesis of cyclotriveratrylenes [4] and oligotoluenes [5,6]—two groups of molecules with attractive chemical properties. Cyclotriveratrylenes (CTVs) [7,8,9,10] are cyclic molecular hosts that have been extensively employed in host–guest chemistry as a supramolecular scaffold [11,12,13,14,15]. CTVs can be prepared in three different ways: (a) by the acid-catalysed condensation of 1,2-disubstituted benzenes with formaldehyde; (b) through the condensation of diphenylmethane with 1,2-disubstituted benzenes and (c) most commonly through the use of dimethoxy-substituted benzyl alcohols under strongly acidic conditions (H_2_SO_4_/CH_3_COOH at 90 °C, H_3_PO_4_ at 80 °C or Sc(OTf)_3_ at 110 °C) [16,17,18,19,20,21]. Oligotoluenes are of industrial interest due to their application as insulating oils in high-voltage electrical devices [22], as reagents in the production of termiticide emulsions that demonstrate good penetration and emulsion stability [23] and as reagents in the preparation of corrosion-protection products [24]. The synthesis of these compounds has been developed following two different strategies: (a) from a mixture of toluene and benzyl chloride under ultrasound (20 MHz, 6 h) and thermal energy conditions (105 °C, 2 h) [5,6] or (b) from benzyl alcohol with carbon disulfide [2] or dichloromethane [3] used as solvents. Both processes were performed in the presence of bentonitic clay.

In recent years, a growth in the use of clays as reaction media for organic transformations has been observed. Natural and modified clays are safer, easy to handle and environmentally attractive compared with mineral acid solutions or metallic catalysts [25,26,27,28,29,30]. Bentonitic clays have been an excellent and versatile acidic Lewis–Brönsted [31] catalyst in the synthesis of Biginelli and Hantzsch esters [32], phenylmethanes [33,34], triphenylbenzenes and triphenylpyrylium salts [35], linear and cyclic ethers [36,37], thiocetals [38], anthracene and its radical cation [39] and in the generation of H_2_O_2_ and hydroxyl radicals [40]. Presently, we desire to extend that knowledge to the development of a process that is more efficient, eliminates the use of solvent and halogenated starting materials, reduces reaction times and avoids tedious workup protocols. With these ideas in mind, we synthesised cycloveratrylene macrocycles and benzyl oligomers from the combination of Tonsil Actisil FF (TAFF), a commercial bentonite clay previously characterized by our group [5,6], with microwave-assisted organic synthesis (MAOS) [41,42,43,44,45] or infrared irradiation [46,47,48,49,50,51,52] in the absence of a solvent. In addition, computational chemistry calculations were performed to establish a validated mechanistic pathway to explain the growth of the obtained linear oligomers.

## 2. Results and Discussion

The initial experiments were performed with alcohols **1a**–**3a** and TAFF under microwave and infrared irradiation; the results are summarised in Table 1. Thus, when compounds **1a**–**3a** were treated with TAFF/MW at 85 °C (100 W) until the disappearance of the starting material after 1.50 min, the corresponding cyclic trimers, namely cyclotripiperonylene (**1b**, CTP, 85%), cyclotriveratrylene (**2b**, CTV, 90%) and 1,2,3,6,7,8,11,12,13-nonamethoxy-10,15-dihydro-5*H*-tribenzo[a,d,g]cyclononene (**3b**, NDTC, 80%), were obtained (Table 1, entries 1 and 3). These molecules are the condensation products of the corresponding benzylic cations. The cyclic trimers should proceed stepwise via the mono-, di- and trimeric cation species and should be followed by a closure step to give the compounds **1b**–**3b** [3].

**Table 1:** Synthesis of cyclovertrylenes ^a^.

Entry		Alcohol		Products	Yield% ^b^		
					MW ^c^		IR ^d^
1		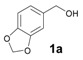		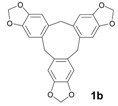	85		80
				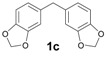	1		3
				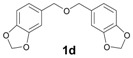	2		5
				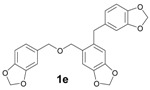	4		5
				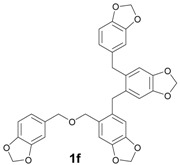	8		7
2		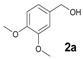		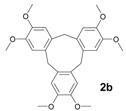	90		88
				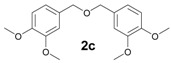	10		12
3	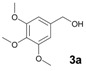		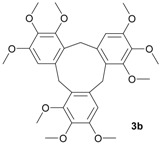		80	75	
			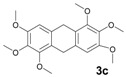		5	5	
			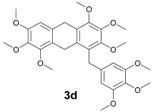		15	20	

^a^ *Reaction conditions*: benzyl alcohols **1a**–**3a** (2 mmol) and TAFF (20 mg); ^b^ Yield of isolated product after chromatographic purification; ^c^ Microwave at 85 °C (100W), 1.50–5 min; ^d^ IR at 95 °C (375W), 3–7 min.

The reactions with alcohols **1a** and **2a** invariably produced a variety of other minor products, which could be a disproportionation product (Table 1, compound **1c**) or are a typical example of an acid-catalysed reaction of a primary alcohol to produce ethers (Table 1, compounds **1d**, **1e**, **2c**). The formation of **1f** might be explained by a subsequent electrophilic aromatic substitution reaction between the ether **1e** and the respective benzyl cation. In the case of alcohol **3a**, 1,2,3,6,7,8,11,12,13-nonamethoxy-10,15-dihydro-5*H*-tribenzo[a,d,g]cyclononene (**3b**) was obtained as the major product, whereas the compounds **3c** and **3d** were the minor products. These products were formed (5% and 15%) as a consequence of the reaction conditions because, when the reaction was performed in carbon disulphide at reflux in the presence of TAFF for 7 h, **3b** was obtained in lesser yield (7%) [36,37].

As shown in Table 1, when the reactions of alcohols **1a**–**3a** were performed under infrared irradiation at 95 °C (375 W), similar results were obtained. The principle difference was the reaction time, which changed for each alcohol. Notwithstanding, a short irradiation time (3–7 min) was required for the complete disappearance of the starting material; this short time was ideal for our goal of developing a time-efficient process.

Next, we considered the possibility of using benzyl alcohols and testing the potential of TAFF/MW and TAFF/IR conditions for inducing the formation of benzyl oligomers. Thus, benzyl alcohol **4a** yielded the oligomers **4c** and **4d** under MW conditions (Entry 1, Table 2). When the reaction was performed using infrared irradiation, oligomers **4c**–**e** were formed (Entry 1, Table 2). In the same context, we also investigated the reactivity of 2-methylbenzyl alcohol (**5a**), which generated benzyl oligomers **5c**–**f** when the reaction was performed under MW conditions and benzyl ethers oligomers **5g**–**i** when the reaction was performed under infrared irradiation (Entry 2, Table 2). Finally, 2-methoxybenzyl alcohol (**6a**) yielded the benzyl oligomers **6c** and **6d** with both MW heating and infrared irradiation (Entry 3, Table 2). In all cases, short irradiation times were necessary for complete reaction of the starting materials (4–10 min).

**Table 2:** Synthesis of oligotoluenes using two different heating models: MW and IR ^a^.

Entry	Alcohol	Products		Yield% ^b^	
				MW ^c^	IR ^d^
1	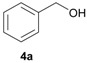	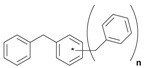	**4b** n = 0	0	0
			**4c** n = 1	60	58
			**4d** n = 2	40	22
			**4e** n = 3	0	20
2	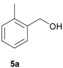	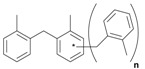	**5b** n = 0	0	0
			**5c** n = 1	55	0
			**5d** n = 2	28	0
			**5e** n = 3	12	0
			**5f** n = 4	5	0
		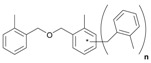	**5g** n = 0	0	65
			**5h** n = 1	0	25
			**5i** n = 2	0	10
3	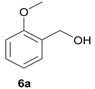	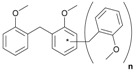	**6b** n = 0	0	0
			**6c** n = 1	35	8
			**6d** n = 2	65	92

^a^ *Reaction conditions*: benzyl alcohols **4a**–**6a** (2 mmol) and TAFF (20 mg); ^b^ Yields and composition of reaction mixture was determinate by GC-EIMS and HRMS; ^c^ Microwave at 85 °C (100 W), 4–10 min; ^d^ IR at 95 °C (375 W), 2.5–10 min.

Given that it is not practical to purify the reaction mixtures by conventional chromatographic methods (CC or HPLC), the presence of these benzyl oligomers in the reaction mixtures was determined by the analysis of GC-EIMS and HRMS spectra following the protocol previously described by our group [5,6]. Thus, when the reaction was analysed by GC-EIMS, each spectrum showed a set of fragments that were assigned unequivocally to the molecular ion of each group of isomers. In addition, each molecular ion was confirmed using high-resolution experiments; consequently, the corresponding elemental composition was obtained (Table 3). Notably, the respective elemental composition change was seven units for carbon and six units for hydrogen; this fact is in agreement with the difference of a benzyl moiety between the groups.

We next considered a theoretical analysis to rationalise the observed products and some of the key reaction steps in the oligomerisation reaction of benzyl alcohol **4a**. We performed a detailed calculation of the molecular structure and electronic properties of the *ortho-*, *meta-* and *para*-trimers, tetramers and pentamers (Table 4) and predicted the relative reactivities and regioselectivities using density functional theory (or Conceptual DFT). We used DFT to analyse the chemical potential, electronegativity, hardness and condensed Fukui functions for an electrophilic aromatic substitution.

**Table 3:** Elemental composition and high resolution data of oligotoluenes.

Alcohol	Products		High resolution	Elemental composition	Yield% ^a^	
					MW	IR
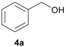	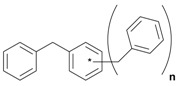	**4c** n = 1	258.1385 ^b^/258.1409 ^c^ (−8.9) ^d^	C_20_H_18_	60	58
		**4d** n = 2	348.1884 ^b^/348.1878 ^c^ (1.6) ^d^	C_27_H_24_	40	22
		**4e** n = 3	438.2355 ^b^/438.2348 ^c^ (1.8) ^d^	C_34_H_30_	0	20
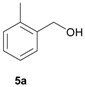	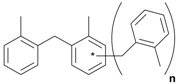	**5c** n = 1	300.1885 ^b^/300.1878 ^c^ (1.8) ^d^	C_23_H_24_	55	0
		**5d** n = 2	404.2498 ^b^/404.2504 ^c^ (1.8) ^d^	C_31_H_32_	28	0
		**5e** n = 3	508.3136 ^b^/508.3130 ^c^ (1.6) ^d^	C_39_H_40_	12	0
		**5f** n = 4	612.3762 ^b^/612.3756 ^c^ (1.6) ^d^	C_47_H_48_	5	0
	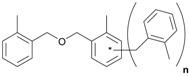	**5g** n = 0	226.1346 ^b^/226.1358 (−5.4) ^d^	C_16_H_18_O	0	65
		**5h** n = 1	330.1990 ^b^/330.1984 ^c^ (1.9) ^d^	C_24_H_26_O	0	25
		**5i** n = 2	434.2614 ^b^/434.2610 ^c^ (1.8) ^d^	C_32_H_34_O	0	10
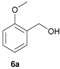	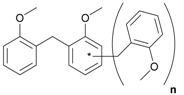	**6c** n = 1	348.1755 ^b^/348.1757 ^c^ (3.8) ^d^	C_23_H_24_O_3_	35	8
		**6d** n = 2	468.2345 ^b^/468.2343 ^c^ (4.6) ^d^	C_31_H_32_O_4_	65	92

^a^ Convertion and composition of reaction mixture was determinate by GC-EIMS and HRMS; ^b^ Observed *m/z*; ^c^ Estimated *m/z*; ^d^ Error (ppm).

**Table 4:** Structure of model compounds: dimer, trimer, tetramer and pentamer.

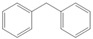	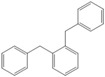	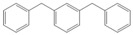	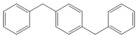
**4b**	**4c-S1o**	**4c-S1m**	**4c-S1p**
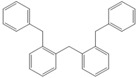	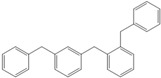	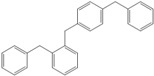	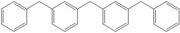
**4d-S1oo**	**4d-S1om**	**4d-S1op**	**4d-S1mm**
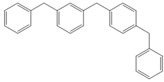	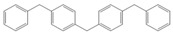	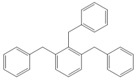	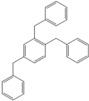
**4d-S1mp**	**4d-S1pp**	**4d-S2om**	**4d-S2op**
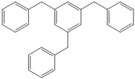	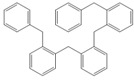	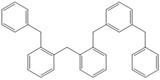	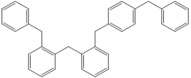
**4d-S2mm**	**4e-S1ooo**	**4e-S1oom**	**4e-S1oop**
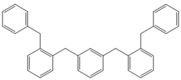	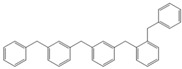	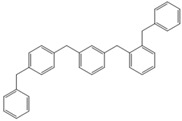	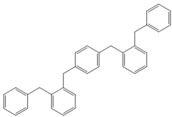
**4e-S1omo**	**4e-S1omm**	**4e-S1omp**	**4e-S1opo**
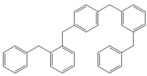	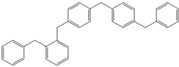	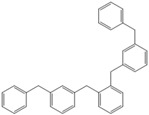	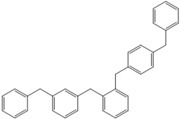
**4e-S1opm**	**4e-S1opp**	**4e-S1mom**	**4e-S1mop**
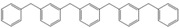	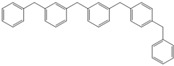	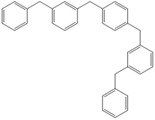	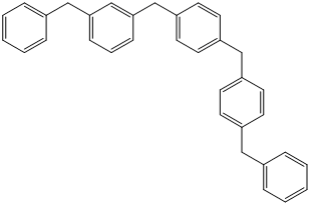
**4e-S1mmm**	**4e-S1mmp**	**4e-S1mpm**	**4e-S1mpp**
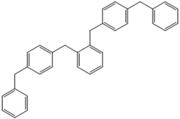	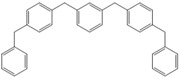	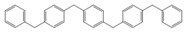	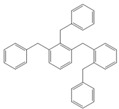
**4e-S1pop**	**4e-S1pmp**	**4e-S1ppp**	**4e-S2ooo**
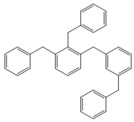	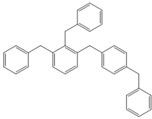	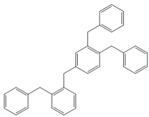	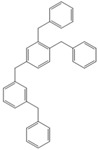
**4e-S2oom**	**4e-S2oop**	**4e-S2omo**	**4e-S2omm**
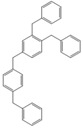	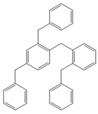	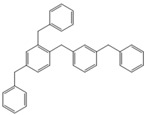	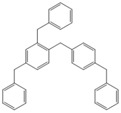
**4e-S2omp**	**4e-S2moo**	**4e-S2mom**	**4e-S2mop**
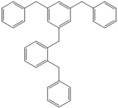	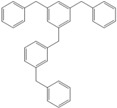	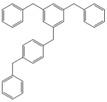	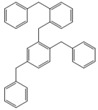
**4e-S2mmo**	**4e-S2mmm**	**4e-S2mmp**	**4e-S2poo**
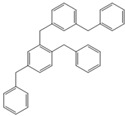	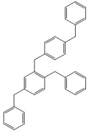	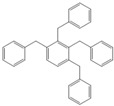	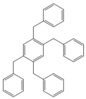
**4e-S2pom**	**4e-S2pop**	**4e-S3oom**	**4e-S3omp**
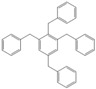	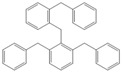	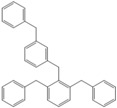	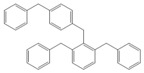
**4e-S3mmp**	**4e-S4moo**	**4e-S4mom**	**4e-S4mop**

**Figure 1:**
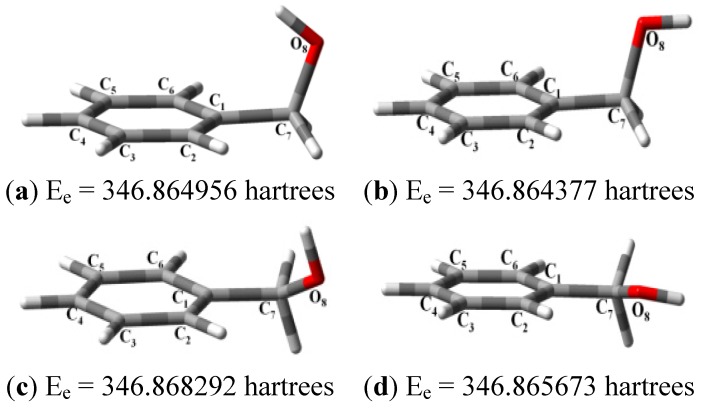
Conformers of benzyl alcohol.

Thus, the geometry of the benzyl alcohol (**4a**) (monomer) was determined using B3LYP/6-311G(d,p). The calculated results of the most stable conformation reproduced the bond distances, C-C_Ar_, within 1.393–1.398 Å; this result is in agreement with the values previously reported for the same compound. Trætteberg and co-workers [53] obtained a d(C-C_Ar_) value of 1.394 Å using the gas electron diffraction method. This molecule has a conformational *gauche* form with the OH group oriented toward the phenyl plane (not stable, Figure 1(a)) and a *trans* form with the OH group oriented away from the phenyl ring (not stable, Figure 1(b)). When the hydrogen atoms on CH_2_ are in *anti* or *syn* orientations to the hydrogen atom of the OH group, the energy difference between the two states is 0.36 kcal/mol. Additionally, the benzyl alcohol has both the *gauche* (Figure 1(c)) and *planar* (Figure 1(d)) orientations of the OH group, but with hydrogen atoms in semiperpendicular orientations toward the phenyl plane. The energy difference between the two states is 1.64 kcal/mol. Both conformational *gauche* forms are stable, but the state with hydrogen atoms in a semiperpendicular orientation toward the phenyl plane is more stable, by 2.09 kcal/mol. The benzyl alcohol monomer in its stable form has dihedral angles CCCO = −32.2° and CCCOH = −58.0°.

The minimum energy molecules of the trimer, tetramer and pentamer can be substituted at the *ortho-*, *meta-* and *para-*positions (Table 4). These systems should exist as different isomers, with each isomer in its respective conformation. The substituted isomers display either a regular or irregular symmetry distribution. All of these structures are stable. The calculated energies of these isomeric compounds are shown in Table 5. Of the three possible isomers of the trimer, the *meta*-isomer appears to be the most stable, followed by the *para*-isomers (the difference between the smallest and greatest is 1.19 kcal/mol). For the nine isomers of the tetramer, *meta*-isomers are more stable than *para*-isomers (the difference between the smallest and greatest is 3.56 kcal/mol). In the case of 39 isomers of the pentamer, the *meta* substitution is more stable than *para*-isomers (the difference between the smallest and greatest is 5.72 kcal/mol). The differences in the relative energies of the isomers of the trimers and tetramers are small and suggest that these isomers are almost identically stable. Additionally, a larger but still relatively small (given that they are conformers) difference in the energy is observed for the pentamers-isomer.

**Table 5:** Computational details.

Structure	E_e_ (Hartrees)	E_rel_ (Kcal/mol)	HOMO (eV)		LUMO (eV)	GAP (eV)	IP (eV)	EA (eV)	Χ (eV)	η (eV)	µ (eV)
**4a**	-	-	−6.81		−0.40	6.41	8.68	−1.66	3.60	5.26	−3.60
**4b**	-	-	−6.54		−0.40	6.14	8.18	−1.17	3.51	4.67	−3.51
**4c-S1o**	−773.166658	1.19	−6.55		−0.52	6.04	8.08	−0.84	3.58	4.43	−3.58
**4c-S1m**	−773.168557	0.00	−6.44		−0.46	5.98	7.83	−0.87	3.48	4.35	−3.48
**4c-S1p**	−773.168276	0.18	−6.36		−0.45	5.92	7.76	−0.87	3.44	4.31	−3.44
**4d-S1oo**	−1043.594698	2.68	−6.50		−0.54	5.96	7.78	−0.68	3.55	4.23	−3.55
**4d-S1om**	−1043.597379	0.99	−6.46		−0.50	5.96	7.73	−0.73	3.86	3.87	−3.86
**4d-S1op**	−1043.596796	1.36	−6.37		−0.52	5.86	7.68	−0.66	3.51	4.17	−3.51
**4d-S1mm**	−1043.598465	0.31	−6.43		−0.53	5.90	7.67	−0.69	3.49	4.18	−3.49
**4d-S1mp**	−1043.598569	0.25	−6.37		−0.50	5.87	7.62	−0.73	3.44	4.18	−3.44
**4d-S1pp**	−1043.598254	0.45	−6.30		−0.46	5.84	7.49	−0.69	3.40	4.09	−3.40
**4d-S2om**	−1043.593291	3.56	−6.51		−0.53	5.97	7.75	−0.68	3.54	4.22	−3.54
**4d-S2op**	−1043.596965	1.25	−6.40		−0.53	5.87	7.70	−0.71	3.50	4.21	−3.50
**4d-S2mm**	−1043.598963	0.00	−6.42		−0.49	5.94	7.69	−0.74	3.47	4.22	−3.47
**4e-S1ooo**	−1314.022344	4.36	−6.47		−0.55	5.92	7.64	−0.58	3.53	4.11	−3.53
**4e-S1oom**	−1314.024711	2.88	−6.37		−0.56	5.81	7.58	−0.57	3.50	4.07	−3.50
**4e-S1oop**	−1314.024876	2.78	−6.38		−0.56	5.82	7.56	−0.56	3.50	4.06	−3.50
**4e-S1omo**	−1314.024522	3.00	−6.51		−0.56	5.95	7.67	−0.56	3.55	4.11	−3.55
**4e-S1omm**	−1314.027076	1.40	−6.44		−0.56	5.88	7.60	−0.62	3.49	4.11	−3.49
**4e-S1omp**	−1314.027660	1.03	−6.36		−-0.53	5.83	7.53	−0.57	3.48	4.05	−3.48
**4e-S1opo**	−1314.024289	3.14	−6.42		−0.55	5.87	7.64	−0.53	3.56	4.08	−3.56
**4e-S1opm**	−1314.027017	1.43	−6.35		−0.55	5.80	7.56	−0.55	3.50	4.05	−3.50
**4e-S1opp**	−1314.026242	1.92	−6.32		−0.51	5.81	7.52	−0.59	3.46	4.05	−3.46
**4e-S1mom**	−1314.027453	1.16	−6.35		−0.54	5.81	7.55	−0.58	3.48	4.07	−3.48
**4e-S1mop**	−1314.027335	1.23	−6.31		−0.49	5.81	7.51	−0.61	3.45	4.06	−3.45
**4e-S1mmm**	−1314.028818	0.30	−6.43		−0.55	5.88	7.58	−0.61	3.48	4.10	−3.48
**4e-S1mmp**	−1314.029167	0.08	−6.33		−0.51	5.82	7.49	−0.61	3.44	4.05	−3.44
**4e-S1mpm**	−1314.028720	0.36	−6.31		−0.50	5.81	7.47	−0.60	3.43	4.03	−3.43
**4e-S1mpp**	−1314.028319	0.62	−6.27		−0.49	5.78	7.39	−0.58	3.41	3.99	−3.41
**4e-S1pop**	−1314.025814	2.19		−6.38	−0.52	5.86	7.48	−0.56	3.46	4.02	−3.46
**4e-S1pmp**	−1314.028888	0.26		−6.29	−0.47	5.82	7.43	−0.65	3.39	4.04	−3.39
**4e-S1ppp**	−1314.028174	0.71		−6.23	−0.47	5.76	7.34	−0.59	3.37	3.96	−3.37
**4e-S2ooo**	−1314.021068	5.17		−6.47	−0.56	5.91	7.63	−0.58	3.52	4.11	−3.52
**4e-S2oom**	−1314.023710	3.51		−6.43	−0.53	5.91	7.58	−0.60	3.49	4.09	−3.49
**4e-S2oop**	−1314.023301	3.76		−6.35	−0.54	5.81	7.53	−0.55	3.49	4.04	−3.49
**4e-S2omo**	−1314.024372	3.09		−6.35	−0.55	5.80	7.61	−0.57	3.52	4.09	−3.52
**4e-S2omm**	−1314.027225	1.30		−6.34	−0.52	5.83	7.54	−0.60	3.47	4.07	−3.47
**4e-S2omp**	−1314.027168	1.34		−6.33	−0.53	5.80	7.51	−0.57	3.47	4.04	−3.47
**4e-S2moo**	−1314.024830	2.81		−6.32	−0.55	5.77	7.55	−0.56	3.50	4.05	−3.50
**4e-S2mom**	−1314.027493	1.13		−6.35	−0.52	5.82	7.53	−0.62	3.45	4.07	−3.45
**4e-S2mop**	−1314.026932	1.49		-6.33	−0.54	5.79	7.48	−0.57	4.46	4.03	−3.46
**4e-S2mmo**	−1314.027206	1.31		-6.35	−0.53	5.82	7.52	−0.60	3.46	4.06	−3.46
**4e-S2mmm**	−1314.029301	0.00		−6.40	−0.56	5.84	7.59	−0.59	3.50	4.09	−3.50
**4e-S2mmp**	−1314.028956	0.22		−6.31	−0.51	5.81	7.50	−0.62	3.44	4.06	−3.44
**4e-S2poo**	−1314.024639	2.92		−6.35	−0.53	5.81	7.57	−0.59	3.49	4.08	−3.49
**4e-Spom**	−1314.027489	1.14		−6.34	−0.51	5.83	7.52	−0.62	3.45	4.07	−3.45
**4e-S2pop**	−1314.026941	1.48		−6.33	−0.54	5.79	7.50	−0.56	3.47	4.03	−3.47
**4e-S3oom**	−1314.020185	5.72		−6.47	−0.58	5.89	7.70	−0.56	3.57	4.13	−3.57
**4e-S3omp**	−1314.024768	2.84		−6.35	−0.60	5.75	7.59	−0.53	3.53	4.06	−3.53
**4e-S3mmp**	−1314.023392	3.71		−6.37	−0.58	5.78	7.57	−0.55	3.51	4.06	−3.51
**4e-S4moo**	−1314.020794	5.34		−6.44	−0.55	5.89	7.62	−0.59	3.51	4.11	−3.51
**4e-S4mom**	−1314.023562	3.60		−6.44	−0.52	5.92	7.61	−0.60	3.50	4.10	−3.50
**4e-S4mop**	−1314.023314	3.76		−6.34	−0.54	5.79	7.52	−0.55	3.48	4.04	−3.48

**Figure 2:**
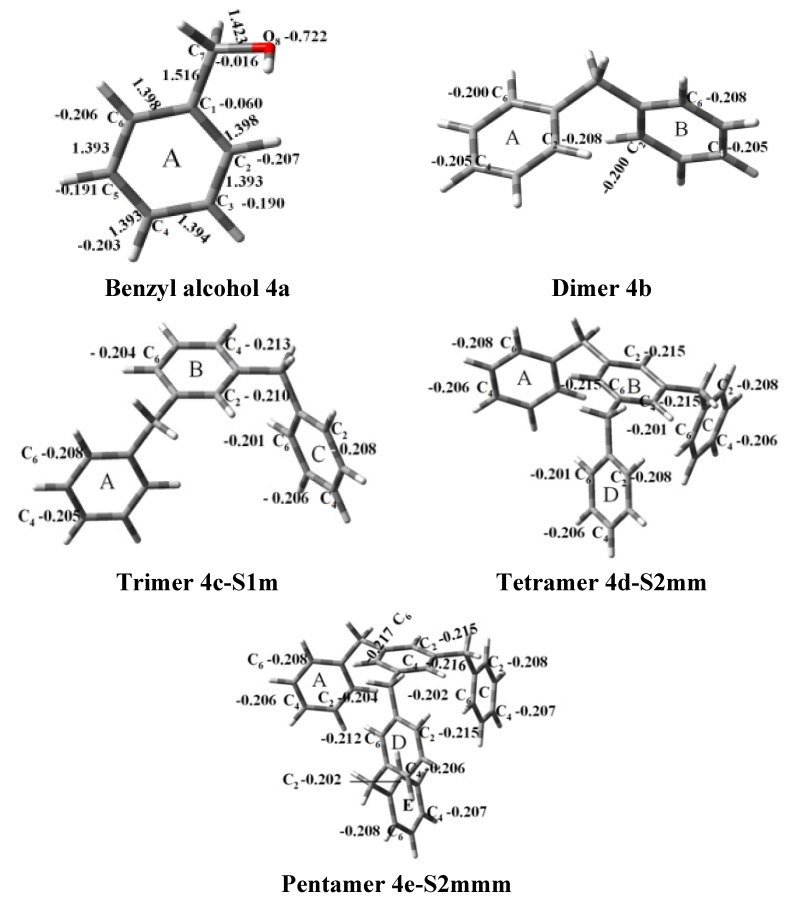
Optimised structures obtained at the B3LYP/6-311G(d,p) level.

Atomic charge values of the dimer, trimer, tetramer and pentamer are depicted in Figure 2. For a particular atom type, differences in its atomic charge can be roughly correlated to differences in its nucleophilic power. The general pattern displayed by NPA [54,55,56,57,58,59,60,61,62,63,64,65,66,67] atomic charges collected in Figure 2 gives the carbon atom in the *ortho* isomer a negative value of approximately −0.208 e, although in this position, steric effects will be present.

**Figure 3:**
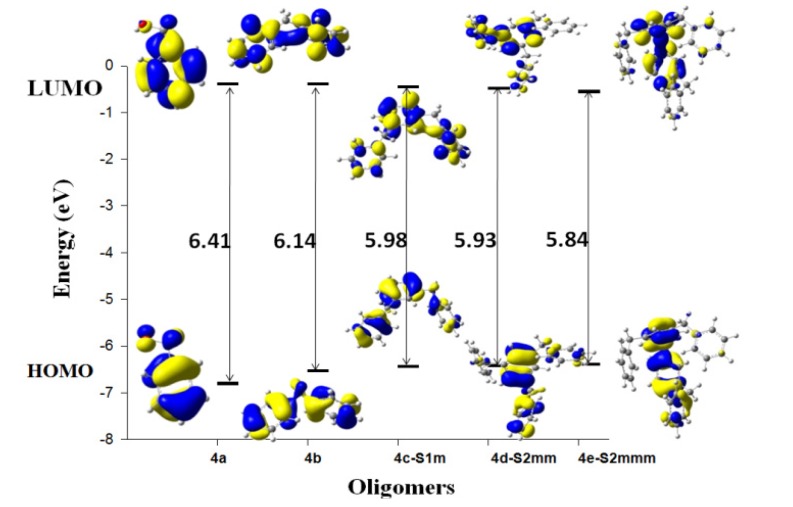
Sketch of B3LYP/6-311G(d,p) calculated energies HOMO, LUMO levels.

**Figure 4:**
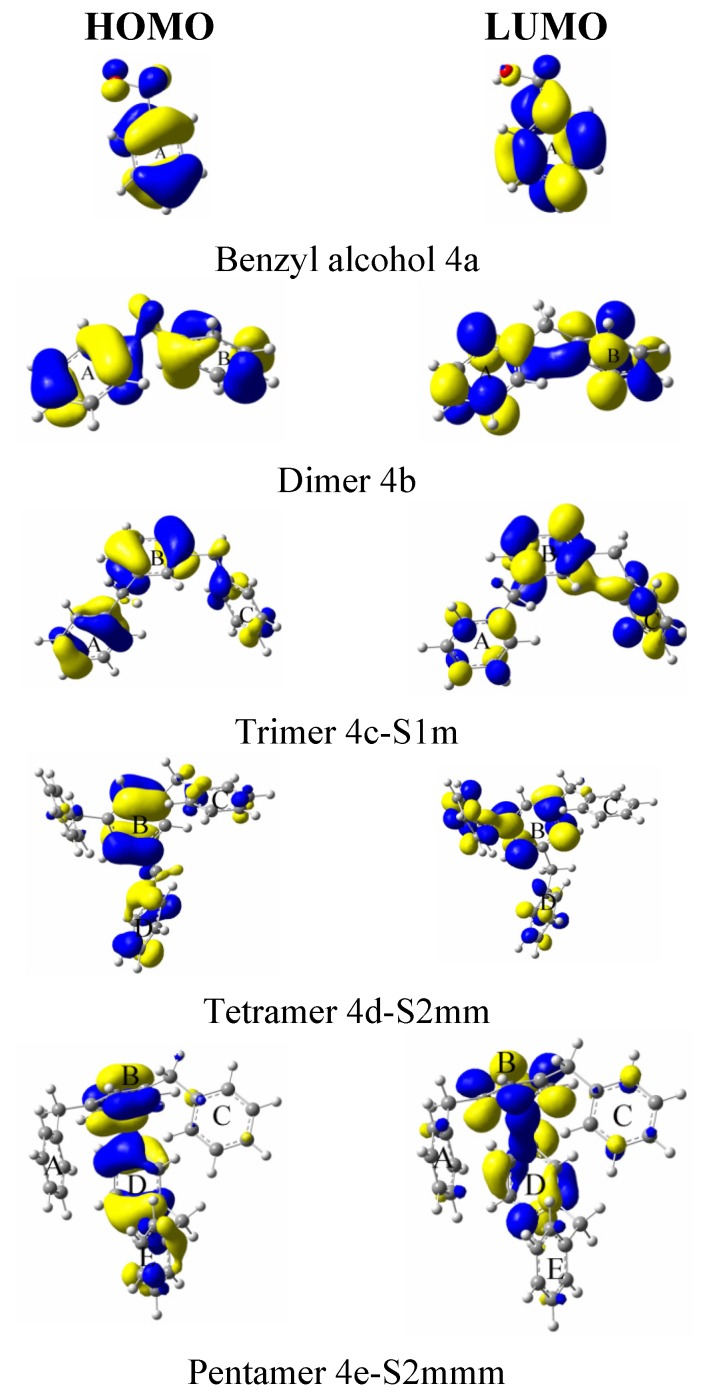
The highest occupied and lowest unoccupied molecular orbitals.

Table 5 shows the HOMO and LUMO energies and the interfrontier molecular orbital energy gaps, ∆E = E_LUMO_ − E_HOMO_, for the considered isomers. The band gap energy values also provide an indication of the stability of a system. For large band gaps, a greater amount of excitation energy is needed to remove an electron from the valence band. From the difference between the smallest and greatest data to the band gap energy values, it is possible to see that the trimer isomers **4c** shows a gap of 0.12 eV, the tetramer isomers **4d** has a value of 0.13 eV, and the pentamer isomers **4e** has a value of 0.2 eV. As evident from the results in Table 5, the *ortho* isomer has the highest HOMO/LUMO gap because of the reduced torsional potential and interaction between the substituted groups. When the substitutions are in the *meta* and *para* positions, the difference in the band gap energy increases with the number of rings; the gap energy is 0.06 eV for the trimer, 0.10 eV for the tetramer and 0.12 eV for the pentamer. Therefore, for an aromatic compound, the HOMO–LUMO gaps must be sufficiently large to prevent electron localisation. This observation is a theoretically correct answer according to the rules of aromaticity—the smaller the HOMO–LUMO band gap, the less aromatic the system. Thus, a linear molecule is a non-aromatic, non-ring structure. If the substitutions are in the *ortho-* and *para-* positions, the difference in the band gap values between them is 0.12 eV for the trimers and tetramers and 0.16 eV for pentamers. The B3LYP/6-311G(d,p)-calculated HOMO, LUMO and gap energies of the studied oligomers are shown in Figure 3. The 4e-S3omp pentamer has the lowest band-gap energy among this group at 5.75 eV, which means that this molecule is the most reactive of all the structures (Table 5). Figure 4 shows the HOMO and LUMO contour molecular orbitals of the more stable oligomers.

The results of the calculations of the reactivity indices, ionisation potential (IP), electron affinity (EA), electronegativity (χ) (as the negative of the chemical potential, µ), chemical potential and hardness (η) for all molecules investigated in this work, as obtained with the B3LYP/6-311G(d,p) model, are presented in Table 5. The ionisation potential of a stable molecule is always positive. An inspection of Table 5 reveals that all molecules present a positive value of the ionisation potential (trimer 7.76–8.01 eV, tetramer 7.49–7.78 eV and pentamer 7.34–7.67 eV). Thus, this result is an indication of the stability of the set of molecules. All of the structures present a negative value for the electron affinity, which indicates that energy is released when a neutral species becomes an anion. The difference between the smallest and greatest energies are −0.84 to 0.87 eV for the trimer, −0.66 to −0.74 eV for the tetramer and −0.53 to −0.62 eV for the pentamer. The released energy decreased with an increase in the number of rings. The monomer has the greatest value of electron affinity, which indicates that the anion is more stable relative to the neutral system. The electronegativity shows how the electrons will flow from regions of high electronic density in a molecule to other sites of lower electronic density. As such, this value is an important index of reactivity for a given system. As evident from the results in Table 5, the electronegativities are given in the following order: 3.6 eV (monomer) > 3.51 eV (dimer) ≈ 3.58–3.44 eV (trimer) ≈ 3.55–3.47 eV (tetramer) ≈ 3.53–3.48 (pentamer). These results indicate that the monomer is more prone to attract electrons during the interaction with another chemical compound. However, we note that the chemical potential of the oligomers derivatives is localised in the following intervals: trimers −3.44 to −3.58 eV, tetramers −3.40 to −3.86 eV and pentamers −3.37 to −3.56 eV. The smallest chemical potential corresponds to substitutions in either *ortho* or *meta* positions; this result clarifies that the flow of charge transfer is from the *para* position to the electrophile during an electrophilic aromatic substitution process. The global hardness of the oligomers, given as the difference between the smallest and largest values, is 4.31–4.43 eV for the trimers, 3.87–4.23 eV for the tetramers and 3.96–4.11 eV for the pentamers.

According to the principle of maximum hardness, more reactive systems will show low hardness values, and less reactive systems will show high hardness values [58]. Molecules arrange themselves to maximise hardness. A high value of chemical hardness indicates high kinetic stability and low reactivity, and, thus, this parameter was found to be a cardinal index for molecular structure, bonding and reactivity. When we consider that the stability of the species is directly related to its hardness, then the stability of the pentasubstituted isomers is lower compared to those of the tetrasubstituted and trisubstituted isomers. Among the trisubstituted, tetrasubstituted and pentasubstituted isomers, the *ortho* isomer was generally found to be the hardest, and the *meta-* and *para*-isomers were the softest. Thus, the hardness measure indicates that the *meta-* and *para*-isomers are the most reactive positional isomers.

**Figure 5:**
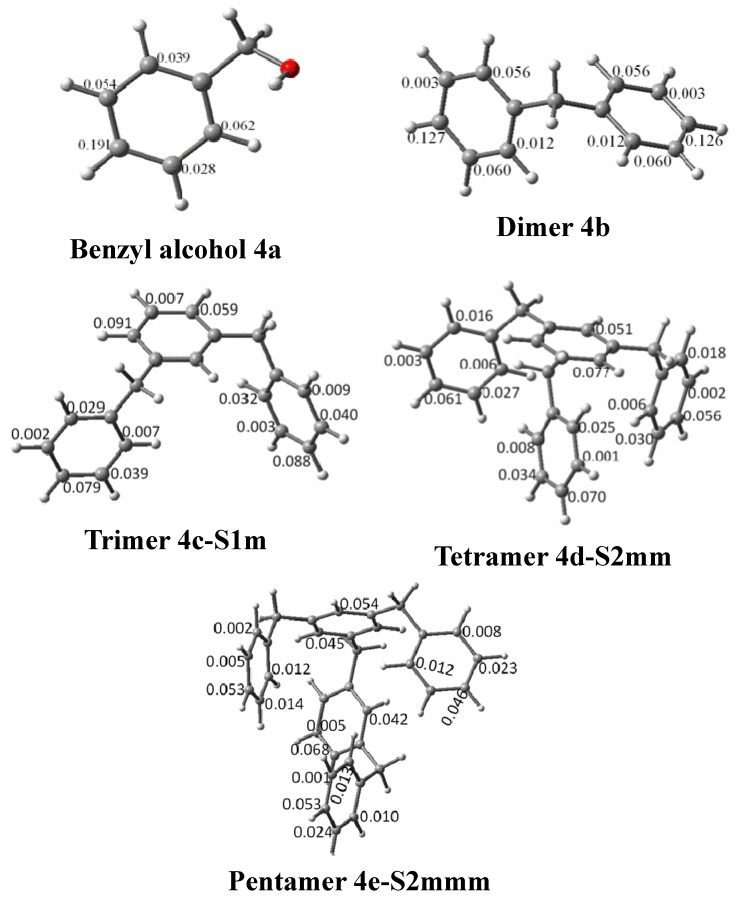
Optimised structures obtained at the B3LYP/6-311G(d,p) level.

To complement the charge analysis and find the active sites of the molecules, we calculated the Fukui functions. The results of the calculations of the condensed Fukui functions, f^–^, for the benzylic alcohol monomers and for some oligomers are presented in Table 6. The benzylic alcohol is more active towards an electrophilic attack through atom Cp (carbon atom in *para*-position). The same atom is inactive for a radical attack or a nucleophilic attack. The dimer has the same activity for either of the two attacks at its *para* carbon atom. The oligomer increases the number of benzylic alcohol units, and its Fukui index decreases. For the other carbon atoms, f^−^ decreased. The charge and HOMO–LUMO molecular orbitals allow us to establish that the growth of the oligomer could occur through the *meta-* position, whereas the Fukui indices indicate that the reactivity is at the *para*-position. According to Figure 5 and Table 6, *para-*carbon in oligomers is the most nucleophilic. 

**Table 6:** Fukui indexe, f^-^, for selected atom, carbon atom, substitution in *para*.

System	Atom ^a^	f^-^
**4a**	Cp	0.191
**4b**	Cp	0.126
	Cp	0.127
**4c-S1p**	Cp	0.079
	Cp	0.078
**4c-S1m**	Cp	0.079
	Cp	0.091
	Cp	0.088
**4c-S1o**	Cp	0.080
	Cp	0.082
	Cp	0.083
	Cp	0.083
**4d-S1pp**	Cp	0.059
	Cp	0.058
**4d-S2om**	Cp	0.051
	Cp	0.056
	Cp	0.065
**4d-S1mp**	Cp	0.062
	Cp	0.070
	Cp	0.058
**4d-S1op**	Cp	0.061
	Cp	0.070
**4e-S1ppp**	Cp	0.044
	Cp	0.044
**4e-S2mop**	Cp	0.050
	Cp	0.056
**4e-S2pop**	Cp	0.045
	Cp	0.057
**4e-S2omp**	Cp	0.046
	Cp	0.051
**4e-S2mpp**	Cp	0.045
	Cp	0.054
	Cp	0.050
**4e-S1opp**	Cp	0.48
	Cp	0.045
**4e-S1pmp**	Cp	0.038
	Cp	0.040
**4e-S1pop**	Cp	0.054
	Cp	0.049

^a^ Cp, *para* substitution in carbon atom.

**Scheme 1:**
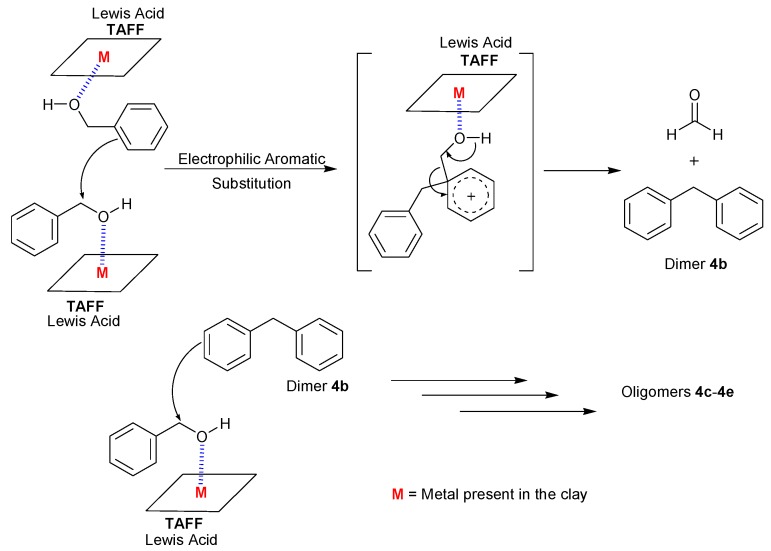
Proposed reaction mechanism for the oligomerisation process.

Finally, from a mechanistic point of view, the catalytic action of TAFF should enhance the electrophilic character of the benzyl alcohol and facilitate the electrophilic aromatic substitution reaction that yields the dimer **4b**, which is the key intermediate to understand the formation of oligomers **4c**–**e**. The interaction between TAFF and benzyl alcohol might be due to the protonated and unprotonated active sites that correspond to the acidic Lewis character of the clay (Scheme 1).

## 3. Experimental

### 3.1. Materials

Tonsil Actisil FF, is cheap (US $1.30/kg) and readily available from Tonsil Mexicana S. A. de C. V. (México City, Mexico). Examined with X-ray fluorescence, this clay was shown to have the following composition (in %): SiO_2_, 74.5; Al_2_O_3_, 9.3; MgO, 0.4; Fe_2_O_3_, 1.3; CaO, 4.0; K_2_O, 0.4; TiO_2_, 0.4; H_2_O, 9.7. X-ray thermodiffractograms show that the laminar structure is unstable above 150 °C. Quartz and cristobalite are also important components in the clay composition, as observed by X-ray diffraction. The corresponding BET surface area is 198.718 m^2^g^−1^, and the pore volume and average pore diameter are 32.04 × 10^−2^ cm^3^g^−1^ and 77.8 Å, respectively. It is worth mentioning that a detailed characterization of the clay (^29^Si and ^27^Al MAS-NMR, SEM, IR-Py, DTA, TG, and Ho) has already been performed and reported by our research group [5,6]. The particle size is 325 mesh. The benzyl alcohols were purchased from Aldrich and were used without further purification. The solvents were also acquired from Aldrich and were purified by standard methods prior to use. All ^1^H- and ^13^C-NMR spectra were recorded on a Varian Gemini (300 MHz) spectrometer using CDCl_3_ as solvent and TMS as an internal reference. EIMS (70 eV) spectra, HRMS data and GC–MS analysis were obtained using a JEOL JMS AX505HA mass spectrometer. The product distribution was determined by a Varian gas chromatograph Stard 3400 equipped with flame ionization detector and a 30m × 0.53 mm column packed with polyethylene glycol, the relative proportion of the products were calculated assuming that the detector gave equal response for each compound. Thin-layer chromatographic analyses were performed using Merck silica gel 60 F_254_ (0.25 mm) pre-coated plates, while products were purified on flash chromatographic columns of silica gel 60 (70−230 mesh). Microwave reactions were performed in a CEM Discover LabMate instrument. The middle infrared irradiation was performed using a Phillips IR lamp (375 W/220 V) integrated to an infrared reactor designed by our research group [46,47,48], and validated by a wide number of applications.

### 3.2. Typical Procedure for the Catalytic Reaction under Microwave Conditions

A mixture of the benzyl alcohols **1a**–**6a** (2 mmol) and Tonsil Actisil FF (20 mg) were reacted in a CEM Discover LabMate in a sealed vessel at 85 °C (100 W and 1 bar of pressure) until disappearance of the starting material. The reaction was conveniently monitored by TLC and the experiments were repeated in various times. Then, the clay was removed by filtration through Celite and washed with ethyl acetate (3 × 5 mL). The combined filtrates were dried on anhydrous Na_2_SO_4_, and the solvent was eliminated under reduced pressure. To the benzyl alcohols **1a**–**3a**, the residue was subjected to chromatography on a silica-gel column using *n*-C_6_H_14_/EtOAc as the eluent, affording the compounds **1b**–**f**, **2b**–**c** and **3b**–**d**. To the benzyl alcohols **4a**–**6a**, the presence of benzyl oligomers in the reaction mixtures was determinate by the analysis of GC-EIMS and HRMS spectra, followed the protocol previously described for our group [5,6]. Table 3 shows the elemental composition and high resolution data of oligotoluenes.

### 3.3. Typical Procedure for the Catalytic Reaction under Infrared Conditions

A mixture of the benzyl alcohols **1a**–**6a** (2 mmol) and Tonsil Actisil FF (20 mg) were thoroughly mixed in a round-bottomed flask (10 mL). The mixtures were exposed to infrared irradiation with an infrared lamp at 95 °C (375 W) until disappearance of the starting material. The reaction was conveniently monitored by TLC. Then, the clay was removed by filtration through Celite and washed with ethyl acetate (3 × 5 mL). The combined filtrates were dried on anhydrous Na_2_SO_4_, and the solvent was eliminated under reduced pressure. To the benzyl alcohols **1a**–**3a**, the residue was subjected to chromatography on a silica-gel column using *n*-C_6_H_14_/EtOAc as the eluent, affording the compounds **1b**–**f**, **2b**–**c** and **3b**–**3d**. To the benzyl alcohols **4a**–**6a**, the presence of benzyl oligomers in the reaction mixtures was determinate by the analysis of GC-EIMS and HRMS spectra (Table 1), followed the protocol previously described for our group. Table 3 shows the elemental composition and high resolution data of oligotoluenes.

### 3.4. Characterization Data

#### 3.4.1. Synthesis of Cyclotripiperonylene (CTP, **1b**)

Following the general procedures, the reaction was carried out starting from 1,3-benzodioxol-5-ylmethanol (**1a**, 304 mg, 2 mmol) and Tonsil Actisil FF (20 mg) under solvent-free conditions. When the reaction was finished (MW = 1.50 min, IR = 3 min) the reaction crude was purified by flash column chromatography on silica gel using *n*-C_6_H_14_/EtOAc to afford the title compound [59]. White solid (MW conditions = 85%, IR conditions = 80%); m.p., decomposes over 300 °C; ^1^H-NMR (CDCl_3_): δ 7.0 (s, 6H, Ar-H), 5.89 (d, 3H, O-CH_2_-O, *J* = 1.0 Hz), 5.77 (d, 3H, O-CH_2_-O, *J* = 1.0 Hz), 4.72 (d, 3H_ax_, Ar-CH_2_-Ar, *J* = 13.7 Hz) 3.45 (d, 3H_eq_, Ar-CH_2_-Ar, *J* = 13.7 Hz); ^13^C-NMR (CDCl_3_): δ 145.3, 132.6, 109.7, 100.8, 36.9; HRMS (FAB) calcd. for C_24_H_18_O_6_ 402.1103, found 402.1098.

In this way, compounds **1c**–**f** were also obtained.

*Compound* **1c** [3]: Yellow oil (MW conditions = 1%, IR conditions = 3%); ^1^H-NMR (CDCl_3_): δ 6.73 (s, 2H, Ar-H), 6.64 (m, 4H, Ar-H), 5.91 (s, 4H, O-CH_2_-O), 3.79 (s, 2H, Ar-CH_2_-Ar); ^13^C-NMR (CDCl_3_): δ 149.3, 146.3, 134.9, 121.6, 115.8, 113.3, 101.2, 46.4; HRMS (FAB) calcd. for C_15_H_12_O_4_ 256.0736, found 256.0732.

*Compound* **1d** [3]: Yellow oil (MW conditions = 2%, IR conditions = 5%); ^1^H-NMR (CDCl_3_): δ 6.86 (s, 2H, Ar-H), 6.79 (m, 4H, Ar-H), 5.95 (s, 4H, O-CH_2_-O), 4.41 (s, 2H, Ar-CH_2_-O); ^13^C-NMR (CDCl_3_): δ 148.7, 147.7, 130.8, 120.8, 115.2, 112.5, 101.2, 74.9; HRMS (FAB) calcd. for C_16_H_14_O_5_ 286.0841, found 286.0836.

*Compound* **1e** [3]: Yellow oil (MW conditions = 4%, IR conditions = 5%); ^1^H-NMR (CDCl_3_): δ 6.87–6.50 (m, 8H, Ar-H), 5.95 (s, 2H, O-CH_2_-O), 5.92 (s, 2H, O-CH_2_-O), 5.90 (s, 2H, O-CH_2_-O), 4.40 (s, 2H, Ar-CH_2_-O), 4.36 (s, 2H, Ar-CH_2_-O), 3.85 (s, 2H, Ar-CH_2_-Ar); ^13^C-NMR (CDCl_3_): δ 149.3, 148.7, 148.4, 147.2, 146.3, 146.2, 134.9, 131.1, 130.8, 121.6, 120.8, 115.8, 115.2, 113.3, 113.2, 113.0, 112.5, 101.2, 74.9, 68.7, 40.2; HRMS (FAB) calcd. for C_24_H_20_O_7_ 420.1209, found 420.1198.

*Compound* **1f** [3]: Yellow oil (MW conditions = 8%, IR conditions = 7%); ^1^H-NMR (CDCl_3_): δ 6.88–6.38 (m, 8H, Ar-H), 5.93 (s, 2H, O-CH_2_-O), 5.92 (s, 2H, O-CH_2_-O), 5.91 (s, 2H, O-CH_2_-O), 5.90 (s, 2H, O-CH_2_-O), 4.35 (s, 2H, Ar-CH_2_-O), 4.27 (s, 2H, Ar-CH_2_-O), 3.78 (s, 2H, Ar-CH_2_-Ar), 3.77 (s, 2H, Ar-CH_2_-Ar); ^13^C-NMR (CDCl_3_): δ 149.3, 148.7, 148.4, 147.9, 146.8, 146.3, 146.2, 134.9, 131.6, 131.1, 130.8, 121.6, 120.8, 115.8, 115.2, 113.8, 113.3, 113.2, 113.0, 112.5, 101.2, 74.9, 68.7, 40.2, 34.0; HRMS (FAB) calcd. for C_32_H_26_O_9_ 554.1577, found 554.1572.

#### 3.4.2. Synthesis of Cyclotriveratrylene (**2b**) [4]

Following the general procedures, the reaction was carried out starting from (3,4-dimethoxyphenyl)methanol **2a** (336 mg, 2 mmol) and Tonsil Actisil FF (20 mg) under solvent-free conditions. When the reaction was finished (MW = 2 min, IR = 4 min) the reaction crude was purified by flash column chromatography on silica gel using *n*-C_6_H_14_/EtOAc. White solid (MW conditions = 90%, IR conditions = 88%); m.p. 231–232 °C; ^1^H-NMR (CDCl_3_): δ 6.83 (s, 6H, Ar-H), 4.78 (d, 3H_ax_, Ar-CH_2_-Ar, *J* = 13.7 Hz), 3.84 (s, 18H, CH_3_-O) 3.56 (d, 3H_eq_, Ar-CH_2_-Ar, *J* = 13.7 Hz); ^13^C-NMR (CDCl_3_): δ 147.9, 132.0, 113.4, 56.2, 36.7; HRMS (FAB) calcd. for C_27_H_30_O_6_ 450.2042, found 450.2038.

In this way, compound **2c** was also obtained.

*Compound* **2c** [3]: Yellow oil (MW conditions = 10%, IR conditions = 12%); ^1^H-NMR (CDCl3): δ 6.91–6.90 (m, 2H, Ar-H), 6.88–6.86 (m, 4H, Ar-H), 4.47 (s, 4, Ar-CH_2_-Ar), 3.84 (s, 18H, CH_3_-O); ^13^C-NMR (CDCl_3_): δ 149.7, 148.9, 130.8, 120.8, 115.2, 112.5, 74.9, 56.2; HRMS (FAB) calcd. for C_18_H_22_O_5_ 318.1467, found 318.1463.

#### 3.4.3. Synthesis of 1,2,3,6,7,8,11,12,13-Nonamethoxy-10,15-dihydro-5H-trbibenzo[a,d,g]cyclononene (**3b**) [60]

Following the general procedures, the reaction was carried out starting from (3,4,5-trimethoxyphenyl)methanol (**3a**, 396 mg, 2 mmol) and Tonsil Actisil FF (20 mg) under solvent-free conditions. When the reaction was finished (MW = 5 min, IR = 7 min) the reaction crude was purified by flash column chromatography on silica gel using *n*-C_6_H_14_/EtOAc. White solid (MW conditions = 80%, IR conditions = 75%); m.p. 199–202 °C; ^1^H-NMR (CDCl_3_): δ 7.24 (s, 3H, Ar-H), 4.42 (d, 3H_ax_, Ar-CH_2_-Ar, *J* = 13.6 Hz) 4.03 (d, 3H_eq_, Ar-CH_2_-Ar, *J* = 13.6 Hz), 3.97 (s, 9H, CH_3_-O), 3.80 (s, 9H, CH_3_-O), 3.77 (s, 9H, CH_3_-O); ^13^C-NMR (CDCl_3_): δ 151.5, 151.4, 140.4, 136.2, 125.5, 110.3, 60.6, 60.5, 55.7, 29.9; HRMS (FAB) calcd. for C_30_H_36_O_9_ 540.2359, found 540.2351.

In this way, compounds **3c**–**d** were also obtained.

*Compound* **3c**: Yellow oil (MW conditions = 5%, IR conditions = 5%); ^1^H-NMR (CDCl_3_): δ 7.06 (s, 2H, Ar-H), 4.57 (s, 4, Ar-CH_2_-Ar), 3.85 (s, 18H, CH_3_-O); ^13^C-NMR (CDCl_3_): δ 148.8, 151.2, 137.2, 132.6, 118.6, 106.1, 56.5, 56.2, 27.1; HRMS (FAB) calcd. for C_20_H_24_O_6_ 360.1573, found 360.1568.

*Compound* **3d**: Yellow oil (MW conditions = 15%, IR conditions = 20%); ^1^H-NMR (CDCl_3_): δ 6.20 (s, 1H, Ar-H), 5.96 (s, 2H, Ar-H), 3.92 (s, 4H, Ar-CH_2_-Ar), 3.80 (s, 2H, Ar-CH_2_-Ar), 3.60 (s, 9H, CH_3_-O), 3.55 (s, 9H, CH_3_-O), 3.50 (s, 9H, CH_3_-O); ^13^C-NMR (CDCl_3_): δ 151.3, 151.2, 148.8, 148.7, 137.7, 137.2, 136.7, 135.9, 132.6, 129.5, 119.1, 106.1, 105.6, 56.5, 56.2, 30.9, 27.4, 20.9; HRMS (FAB) calcd. for C_30_H_36_O_9_ 540.2359, found 540.2354.

#### 3.4.4. Synthesis of Oligotoluenes **4c**–**e**

Following the general procedures, the reaction was carried out starting from phenylmethanol (**4a**, 216 mg, 2 mmol) and Tonsil Actisil FF (20 mg) under solvent-free conditions. When the reaction was finished (MW = 10 min, IR = 10 min) the presence of benzyl oligomers **4c**–**e** in the reaction mixture was determined by GC-EIMS analysis and HRMS spectroscopy.

*Synthesis of Oligotoluenes* **5c**–**i**: Following the general procedures, the reaction was carried out starting from (2-methylphenyl)methanol (**5a**, 244 mg, 2 mmol) and Tonsil Actisil FF (20 mg) under solvent-free conditions. When the reaction was finished (MW = 5 min, IR = 7.5 min) the presence of benzyl oligomers **5c**–**i** in the reaction mixture was determined by GC-EIMS analysis and HRMS spectroscopy.

*Synthesis of Oligotoluenes* **6c**–**d**: Following the general procedures, the reaction was carried out starting from (2-methoxylphenyl)methanol (**6a**, 276 mg, 2 mmol) and Tonsil Actisil FF (20 mg) under solvent-free conditions. When the reaction was finished (MW = 4 min, IR = 5 min) the presence of benzyl oligomers **6c**–**d** in the reaction mixture was determinate by GC-EIMS analysis and HRMS spectroscopy (Table 3).

### 3.5. Theoretical Calculations

Calculations were performed using the Gaussian 09 system [61]. The geometries of the isolated benzyl alcohol molecule and its dimer, trimers, tetramers and pentamers complexes were fully optimized at the B3LYP [62,63] level using the 6-311G(d,p) basis set [64,65]. Harmonic vibrational frequency calculations have been performed at the same computational level to confirm that the structures are energetic minima. In the present work we analyze the theoretical structure and energetic of the *ortho*, *meta*, and *para* trimmers, tetramers and pentamers (Table 4). Natural population analyses (NPA) were also performed based on the optimized geometry [66]. We have also examined HOMO and LUMO levels; the energy gap is evaluated as the difference between the HOMO and LUMO energies. The quantitative definitions for chemical potential (µ) [67] and chemical hardness (η) [68,69] for an N-electron system with total energy E can respectively be given as:

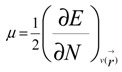
(1)
and:

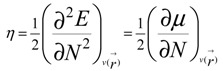
 (2)
where 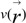 is the external potential. Using a finite difference method the working equations for the calculation of chemical potential and chemical hardness can be given by:

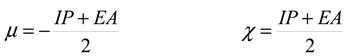
(3)

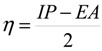
(4)
where IP and EA are ionization potential and electron affinity of the system, respectively. Using the ∆SCF finite difference approach, we can calculate the IP and EA for the N-electron system as follows:

Ionization potential (*IP*)    *IP* = E(N − 1) − E(N)

Electron affinities (*EA*)    *EA* = E(N) − E(N + 1)

kui functions are common descriptors of site reactivity [70,71,72]. They are defined as the derivative of the electron density with respect to the total number of electrons N in the system, at the constant external potential.

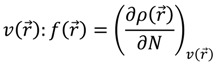
(5)
The condensed Fukui functions can also be employed to determine the reactivity of each atom in the molecule. The corresponding condensed functions are given by:

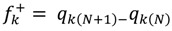
   (for nucleophilic attack)(6)

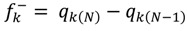
   (for electrophilic attack), and (7)

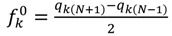
   (for radical attack), (8)
where *q_k_* is the gross charge of atom *k* in the molecule. A high value of *f_k_* implies a high reactivity of that site *k*. Besides, the type of condensed Fukui function whose value is highest at a particular site, predicts the type of attack that predominates at that site.

## 4. Conclusions

In summary, we have developed an efficient and solvent-free protocol for the synthesis of cycloveratrylene macrocycles and benzyl oligomers under mild conditions in the presence of Tonsil Actisil FF (TAFF), a commercial bentonite clay, under microwave heating and infrared irradiation. Compared with the traditional method, this method possesses at least four advantages: (i) the energy supply (MW and IR) is harmless; (ii) the reaction was performed in the absence of carbon disulphide or dichloromethane, which means that no volatile organic compounds (VOCs) were used in the present protocol; (iii) the reaction times are short; and (iv) the use of any halogenated starting materials, metal compounds or corrosive acids that may cause pollution are avoided. The only catalyst used in this reaction is bentonite clay, which can be easily removed from the reaction mixture. Notably, the catalytic reaction is sensitive to the type of substituent on the aromatic ring. Thus, when benzyl alcohol was substituted with a methylenedioxy, two methoxy or three methoxy groups, a cyclooligomerisation process was induced. Unsubstituted, methyl- and methoxybenzyl alcohols yielded linear oligomers.

In addition, to predict the relative reactivities and regioselectivities of the oligomerisation processes of benzyl alcohol, we calculated the energies and optimised the structures of trimer, tetramer and pentamer isomers. The *meta*-isomer appears to be more stable than the *para*-isomers. After the reactivity descriptors calculated for the oligomer derivatives were analysed to identify the system with the lowest band gap and the largest ionisation potential, we calculated these values for the penta-oligomers. The penta-oligomers also showed a lower electron affinity. The chemical hardness indicates that as the number of benzylic alcohol units is increased, the oligomer becomes less stable. These molecules are more reactive. Additionally, the position of substitution is important. The electronegativity of the oligomer derivatives is almost the same. Finally, the charge indicates that reactivity is highest at the *ortho* position but also that steric effects are present. The HOMO–LUMO energy gap allows us to establish that the growth of the oligomer could occur at the *meta-* or *para-* position. The Fukui indices, however, indicate that the highest reactivity is at the *para*-position.
